# An individual approach to feline diabetes care: a case report and literature review

**DOI:** 10.1186/s13028-016-0245-0

**Published:** 2016-10-20

**Authors:** Moira S. Lewitt, Emma Strage, David Church

**Affiliations:** 1 School of Health, Nursing and Midwifery, University of the West of Scotland, Paisley, Scotland, UK; 2 Department of Clinical Sciences, Swedish University of Agricultural Sciences, Uppsala, Sweden; 3 Department of Veterinary Clinical Sciences, Royal Veterinary College, University of London, North Mymms, Hertfordshire, UK

**Keywords:** Diabetes mellitus, Remission, Insulin therapy, Home glucose monitoring

## Abstract

**Background:**

Achieving insulin independence is emerging as a realistic therapeutic goal in the management of feline diabetes mellitus.

**Case presentation:**

The management of an 11-year-old spayed female Burmese cat presenting with diabetes mellitus after corticosteroid administration is described. Remission was achieved after the frequency of insulin administration was increased to four times a day, and supported by intensive home blood glucose monitoring and a high protein, low carbohydrate diet.

**Conclusion:**

Owners are important collaborators in feline diabetes care and, with intensive home monitoring, more frequent insulin treatment may lead to remission without hypoglycemia. More frequent insulin injections than recommended in the literature may be necessary to achieve glycemic control and used as an alternative to a longer-acting insulin.

## Background

Feline diabetes mellitus is characterised by insulin resistance and impaired insulin secretion, and its prevalence is increasing, partly due to lifestyle factors, including indoor confinement and physical inactivity [1–3]. Lean cats with low insulin sensitivity are predisposed to impaired glucose tolerance with weight gain [4]. Increasing weight is a risk factor for diabetes [1, 3, 5], along with advancing age [1, 5, 6] and sex, with diabetes being more common in neutered male cats [1, 3, 5, 6]. Burmese cats in the UK are 3.7 times more likely to develop diabetes than non-pedigree cats [3]. Similar observations have been made of Burmese cats in Australia [7, 8] and Sweden [9]; and recent studies suggest that this is due to dysregulation of lipid metabolism in this breed [10, 11]. A recent pilot study suggests involvement of a major gene locus in diabetes in Burmese cats [12].

Diabetes remission rates exceeding 60 % have been reported in cats receiving pharmacological and dietary management [13–15], and remission may be achieved in cats that have ketoacidosis at presentation [16]. Insulin and diet are the mainstays of therapy. It is reported that the remission rate is higher on glargine compared to porcine lente insulin [17]. A recent systematic review has reported that successful remission rates vary considerably and that remission is associated with a variety of insulin types and protocols [18]. The duration of insulin action of glargine, although reported to be on average longer than lente [19], may vary from animal to animal [20]. There is an anecdotal report of effective management in two diabetic cats using multiple daily injections of a mixture of short acting and long acting insulin [21].

While insulin independence is a realistic therapeutic goal in feline diabetes, remission is dependent on achieving glycemic control quickly [22] and, without intensive monitoring, high insulin doses increase the risk of hypoglycemia. We present a case of an 11-year old spayed Burmese cat that presented with diabetes after steroid treatment for skin allergy. Control of blood glucose was not achieved using a low carbohydrate diet plus the recommended twice-daily treatment with either insulin lente or glargine, with the total dose limited by the risk of hypoglycemia. A more frequent insulin regimen using glargine was used successfully.

## Case presentation

**Fig. 1:**
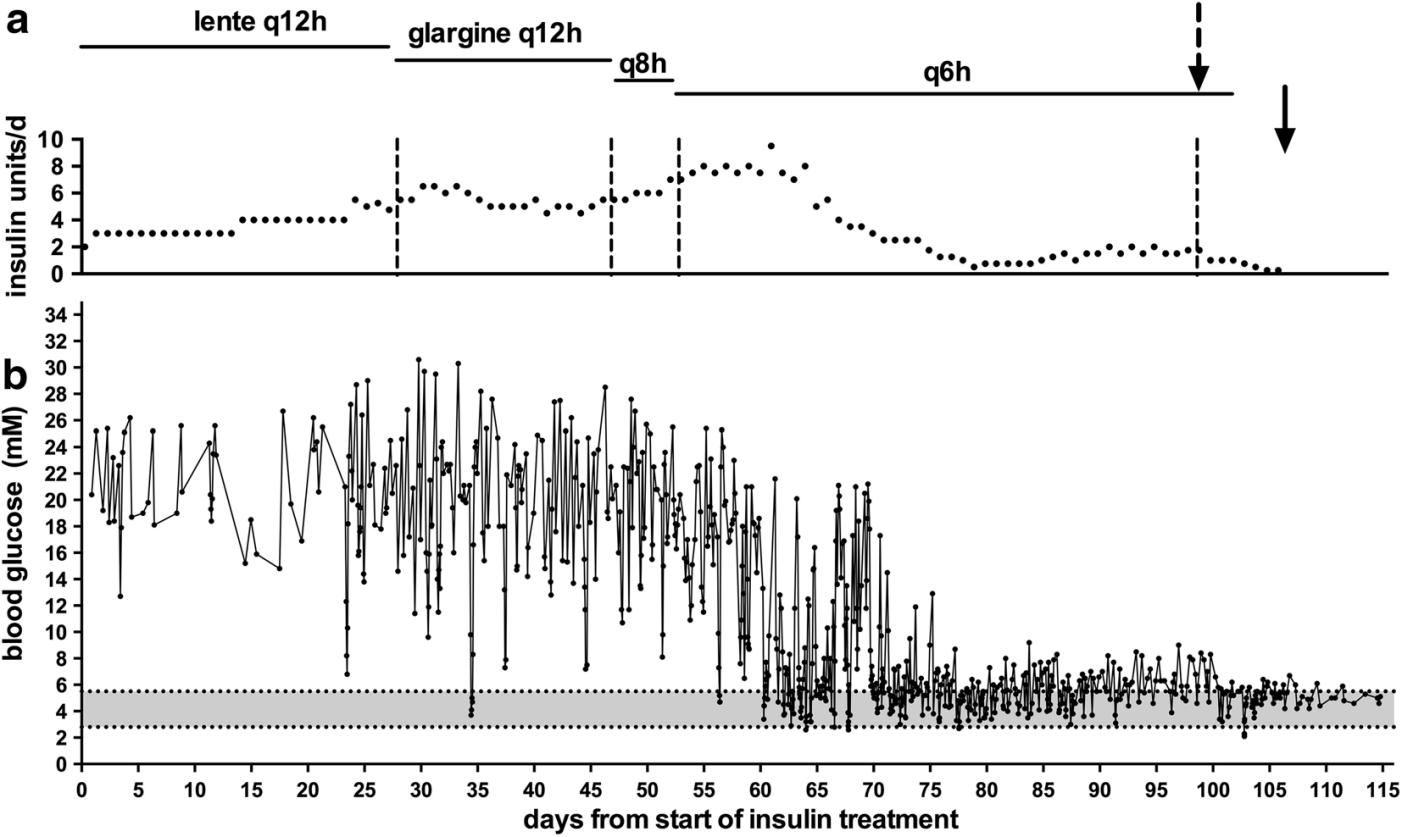
Total daily insulin doses (**a**) and all blood glucose values (**b**) from commencement of treatment until 1 week after cessation of insulin. The *shaded area* represents the normal blood glucose range. The *broken arrow* represents the time that caloric intake was restricted to a maximum of 75 g wet and 25 g dry food (Purina DM) and increased physical activity by encouraging play several times a day. The *solid arrow* indicates the time when insulin was ceased

A five-year-old female neutered Burmese (Swedish and UK origin) was started on a management strategy for allergic dermatitis consisting of short course of 5 mg prednisolone twice daily, rapidly tapering and withdrawn after 3 weeks. This programme was repeated five times over the next 6 years until at 11 years of age the cat received a single injection of methylprednisolone (Depo-Medrol 20 mg i.m.) and, within 5 days, was observed to have polydipsia and polyuria. Home urinalysis (Keto-Diastix, Bayer) revealed glucose (2+) without ketonuria and, at initial veterinary assessment 2 days later the cat weighed 3.2 kg (last recorded weight was 3.5 kg 18 months previously) and had a body condition score of 4 (on a 9 point scale [23]) with no other significant abnormalities detected on physical examination. Routine serum biochemistry revealed marked hyperglycemia (blood glucose concentration 29.8 mmol/L (reference range 3.9–8.8) and increased fructosamine concentrations (481 µmol/L, 190–340). All other measured parameters were within normal limits. Initial management consisted of a high protein, low carbohydrate diet (Purina DM wet and dry food, fed ad libitum in a ratio of at least 3:1) and twice daily porcine lente insulin (Caninsulin, MSD Animal Health), starting with 1 unit q12 h, started immediately (on day seven after the injection of methylprednisolone). Capillary blood monitoring from the pinna of the ear was commenced using a blood glucose meter calibrated for human blood that is used in cats (Accu-Chek Aviva, Roche UK; feline reference range 2.8–5.5 mmol/L for meter [14]). Blood glucose was taken prior to insulin injection. On some days glucose was also measured more frequently between insulin injections; for example every 3 h or when hypoglycaemia was suspected). Figure 1 shows all of the results for blood glucose testing for the first 4 months of management. During the first 7 days of testing the mean glucose value was 21 mmol/L. The owner obtained a urine sample, which was delivered to the local veterinary practice, and which revealed a pure growth of an Enterococcus. Given the clinical circumstances, this was suspicious of UTI and was treated with antibiotics by the veterinary surgeon.

**Fig. 2:**
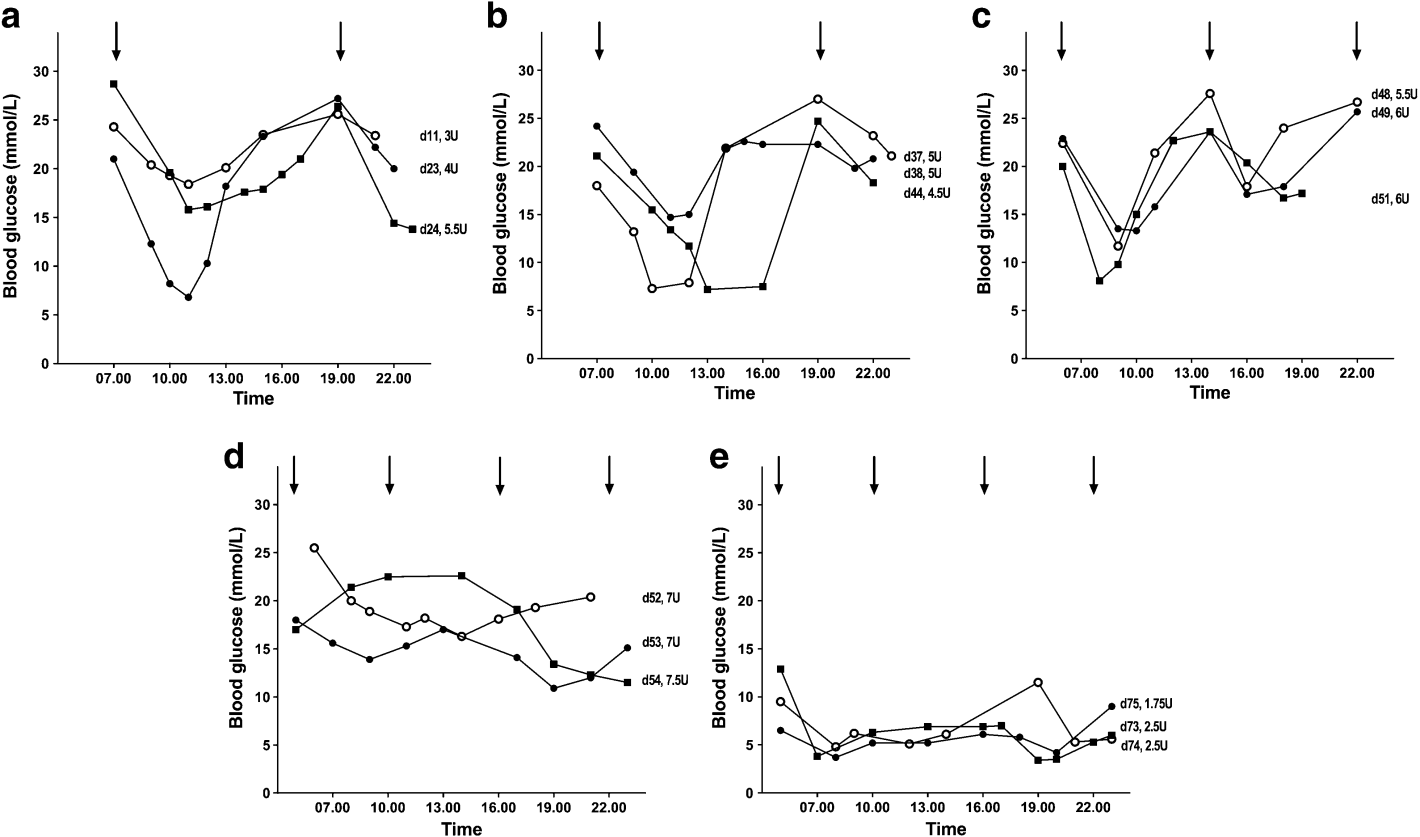
Daily blood glucose profiles on porcine lente insulin 12-hourly (**a**) and glargine 12-hourly (**b**), 8-hourly (**c**) and 6-hourly (**d**, **e**). Times of insulin administration are indicated by the *arrows*. The day from the start of insulin treatment and total daily units (U) of insulin are indicated to the right of each profile

**Fig. 3:**
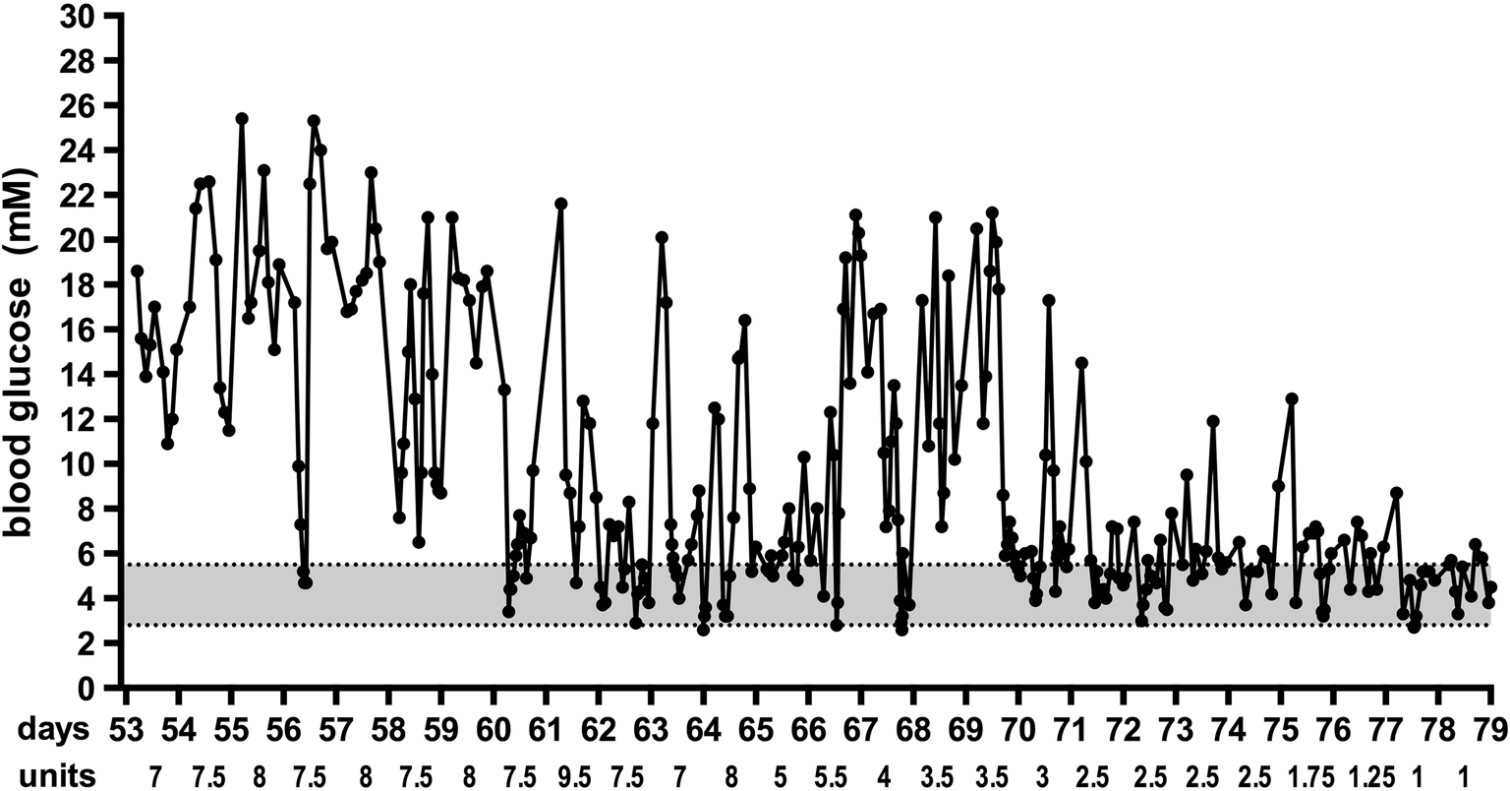
All blood glucose values from day 53 to day 79 of treatment. The *shaded area* represents the normal blood glucose range. The total daily glargine dose, that was divided and delivered 6-hourly, is shown

On day 27, the cat was on porcine lente insulin, 2.5 units insulin q12 h and glucose curves documented clear falls in glucose in response to injections, with a nadir at around 4 h (Fig. 2a). It was considered that the short duration of insulin action might be a limiting factor in achieving good glycemic control and the cat was started on glargine, an insulin analogue registered for human use. On q12 h glargine (4.5–6.5 units per day; 2–3.5 units per injection; Lantus, Sanofi-Aventis) there appeared to be no difference in the pattern of glucose responses (Fig. 2b), again limiting the total daily dose of insulin that could be delivered without an unacceptable risk of hypoglycemia. For this reason and, concerned that the chance of β cell recovery was diminishing with increasing duration of hyperglycemia, on day 47 a decision was made to increase the frequency of glargine injections to 8-hourly, with a slight increase total daily dose of insulin (to 5.5–7 units per day; 1.5–2.5 units per injection). The owner performed frequent blood glucose measurements at home (Fig. 2c). On day 53 the frequency of injections was increased to 6-hourly, with an increase in the total dose (to 7–9 units per day; 1.5–3 units per injection) (Fig. 2d, e) and the daily insulin dose was tapered (Fig. 3) from 2 units q6 h on day 64 to 0.25 units q6 h on day 77. Intensive monitoring, often every three hours, on day 67–70 revealed that the lowest blood glucose value was 2.7 mmol/L on days 64 and 67. Although only one blood glucose value of <2.8 mmol/L had been recorded, the owner-physician interpreted the relative hyperglycemia during this period to represent rebound hyperglycaemia, and continued to taper the dose of insulin. On day 62 and 65, the dose of insulin was reduced by 21 and 37 %, respectively; the decrease in dose over this time was otherwise not greater than 20 %. On days 77 and 102, blood glucose levels of 2.7 and 2.1, respectively were recorded and were not followed by hyperglycaemia.

From day 77, when the cat was on a total daily dose of 1 unit of glargine, glucose concentrations were mostly within the reference range. However it seemed that insulin was still required to achieve euglycemia, and that requirements to achieve euglycaemia were increasing. On day 95, on 0.5 units q6 h glargine, glucose concentrations were 6–8 mmol/L and a fructosamine concentration was 280 µmol/L, in the middle of the reference range. On day 98 it was noted that the weight had increased to 3.9 kg with a body condition score of 6. The total caloric intake was thereafter restricted to a maximum of 75 g wet and 25 g dry food (Purina DM) and physical activity was increased by playing with the cat several times a day. Within 5 days insulin was withdrawn and 1 month later, a visit was made to the local veterinary surgeon. The fructosamine was 271 µmol/L. Over the following 4 years later the cat has remained insulin-independent and, when glucose concentrations are measured (occasionally) these ranged from 4.6 to 5.1 mmol/L.

## Conclusions

This case demonstrates that changing the frequency of insulin injections can be considered as an alternative to changing to a longer-acting insulin and offered to owners of cats not achieving control on twice daily regimes. Hypoglycemia is one of the biggest concerns for owners of cats with diabetes [24] and is associated with larger doses of insulin [25]. It is possible that the risk of hypoglycemia could be avoided by dividing the insulin dosage and administering at more frequent intervals. Alternatively some cats may respond to changing to an insulin that is longer-acting, for example changing from lente to glargine, or from glargine to detemir [26]. These insulins may have a shorter duration of action than in humans [20]. Detemir has less variability in duration of action between cats and in some cats, the duration of action is longer than for glargine [20]. The phase of relative hyperglycemia observed in this case report was interpreted by the owner as representing a significant counter-regulatory response, even in the absence of documented hypoglycemia, and responded by reducing the insulin dose. In retrospect, looking at the pattern of blood glucose levels and considering the intensive monitoring, it is unlikely that frank hypoglycaemia was responsible for the paradoxical increase in blood glucose. An alternative explanation is that decreases in insulin dose may have contributed to the phenomenon.

The decline in β cell function in feline diabetes is believed to be a consequence of glucotoxity, that is initially reversible but later irreversible [18]. Glucocorticoid administration is a well-documented risk factor for feline diabetes and may well have contributed to the onset of hyperglycemia by decreasing insulin sensitivity in this case. Cats treated with corticosteroid in the 6 months prior to diabetes diagnosis are more likely to go into remission than those that did not receive steroids [14]. In this case the product delivered was an insoluble steroid ester and hence its effect could well have lasted for up to 6 weeks. However, once insulin resistance had improved, appropriate insulin dosing appeared to be important to resolve glucotoxicity for recovery of β cell function. Other reported risk factors were also present in this cat, including advancing age, indoor confinement and physical inactivity, and Burmese breed [7–9]. Although diets that are low in carbohydrate have an important place in management of the disease [13, 27], when fed ad libitum they may lead to greater weight gain than diets that are relatively high in carbohydrate and low in fat [28], and may have contributed to the weight gain in this case because they are also high in fat [29].

The owner of this particular case, a physician herself, took a great deal of responsibility in day-to-day management decisions. A recent feline diabetes-associated quality of life survey indicated that the areas reported as most negatively impacting included “owner wanting more control” [24]. In humans, interventions that involve patient collaboration are particularly important in improving self-care behaviours, quality of life and empowerment, to such an extent that the International Diabetes Federation has deemed that diabetes self-management education is considered a right for all those with, or at risk of, diabetes [30]. It is similarly recommended that owners of cats with diabetes should be informed of the options available to them, including the benefits of tight glucose control and home capillary glucose monitoring.
